# Supplementary material for: AlphaFold-Multimer modelling of linked nAChR subunits challenges concatemer design assumptions
Source: Sci Rep. 2026 Apr 27;16:19470. doi: 10.1038/s41598-026-50253-3 (PMC13287450; doi:10.1038/s41598-026-50253-3)
Supplement: Supplementary file 1 — Supplementary Material 1 [file 41598_2026_50253_MOESM1_ESM.docx]

**Table S1.** Names and pedigree of the six durum wheat genotypes used in this study.

| **Name** | **Pedigree** |
| --- | --- |
| Wt | Tunisian wild-type durum wheat cultivar Karim |
| Lines (K9.3, K21.3) | T3 homozygous genetically engineered lines expressing low and high levels of AlSAP, respectively, obtained from Dr. Afif Hassairi, described by Ben Saad et al. |
| Line KSU101 | Derived from the cross (Line-14 × Benysowef), obtained from |
| Line KSU102 | Derived from the cross (Line-118 × Benysowef) |
| Line KSU104 | Derived from the cross (Sham1 × Benysowef) |

**Table S2.** Monthly agro-climatological data at the experimental location during the two growing seasons.

| Parameters  Months | Precipitation (mm) | | Temperature (°C) | | | | | | Relative Humidity (%) | | |
| --- | --- | --- | --- | --- | --- | --- | --- | --- | --- | --- | --- |
|  |  |  | Maximum | | Minimum | | Average | |  |  |  |
|  | S1 | S2 | S1 | S2 | S1 | S2 | S1 | S2 | S1 | S2 |  |
| November | 0.31 | 0.27 | 35.37 | 34.82 | 5.05 | 5.84 | 20.21 | 20.33 | 41.69 | 41.21 |  |
| December | 0.02 | 0.10 | 27.40 | 27.46 | 4.01 | 4.95 | 15.71 | 16.21 | 45.31 | 44.36 |  |
| January | 0.10 | 0.05 | 29.34 | 28.65 | 1.23 | 2.01 | 15.29 | 15.33 | 40.19 | 40.68 |  |
| February | 0.00 | 0.00 | 33.79 | 32.43 | 1.21 | 2.08 | 17.50 | 17.26 | 28.44 | 29.26 |  |
| March | 0.00 | 0.00 | 35.80 | 36.19 | 6.98 | 7.01 | 21.39 | 21.60 | 25.69 | 26.02 |  |
| April | 0.98 | 1.02 | 39.91 | 38.63 | 14.13 | 15.11 | 27.02 | 26.87 | 31.50 | 30.24 |  |
| May | 0.01 | 0.00 | 42.91 | 41.77 | 19.21 | 19.94 | 31.06 | 30.86 | 17.69 | 17.05 |  |

S1, Season 2018/2019; S2, Season 2019/2020.

**Table S3**. Results for WAASB estimation of six genotypes assessed in six environmental environments (combinations of two years)

| **type** | **Code** | **Y** | **PC1** | **PC2** | **PC3** | **PC4** | **PC5** | **WAAS** | **WAASY** |
| --- | --- | --- | --- | --- | --- | --- | --- | --- | --- |
| GEN | K21.3 | 4.54 | -0.247 | -0.571 | -0.193 | -0.0245 | -6.7E-09 | 0.296 | 83.5 |
| GEN | K9.3 | 4.42 | 0.0547 | -0.0633 | 0.0858 | 0.178 | -7.11E-09 | 0.056 | 92.4 |
| GEN | KSU101 | 4.21 | 0.154 | 0.435 | -0.207 | 0.0132 | -3.49E-09 | 0.198 | 69.8 |
| GEN | KSU102 | 3.88 | -0.085 | 0.304 | -0.00849 | -0.0538 | -1.11E-08 | 0.119 | 54.7 |
| GEN | KSU104 | 3.82 | -0.775 | 0.0503 | 0.186 | -0.0471 | -4.1E-09 | 0.664 | 13.7 |
| GEN | Wt | 3.73 | 0.898 | -0.156 | 0.137 | -0.0656 | -5.4E-09 | 0.784 | 0.00 |
| ENV | CS1 | 5.39 | 0.602 | 0.433 | 0.191 | 0.0403 | -3.98E-09 | 0.576 | 55.2 |
| ENV | CS2 | 5.88 | 0.787 | -0.259 | -0.202 | -0.0323 | -3.98E-09 | 0.706 | 50 |
| ENV | DS1 | 3.06 | -0.472 | 0.4 | -0.126 | -0.0803 | -9.49E-09 | 0.461 | 26.2 |
| ENV | DS2 | 3.45 | -0.199 | -0.466 | 0.194 | -0.0364 | -9.49E-09 | 0.24 | 56.9 |
| ENV | HS1 | 3.25 | -0.336 | -0.0328 | 0.0342 | -0.0584 | 7.04E-09 | 0.289 | 48.2 |
| ENV | HS2 | 3.58 | -0.381 | -0.0751 | -0.0914 | 0.167 | -3.98E-09 | 0.334 | 49.1 |
